# Half back stitch for root reimplantation

**DOI:** 10.1016/j.xjtc.2024.02.017

**Published:** 2024-04-25

**Authors:** Wan Kee Kim, MinJung Ku, Hong Rae Kim, Joon Bum Kim

**Affiliations:** a Department of Thoracic and Cardiovascular Surgery, Hanyang University Seoul Hospital, Hanyang University College of Medicine, Seoul, Republic of Korea; b Department of Thoracic and Cardiovascular Surgery, Asan Medical Center, University of Ulsan College of Medicine, Seoul, Republic of Korea

**Figure:**
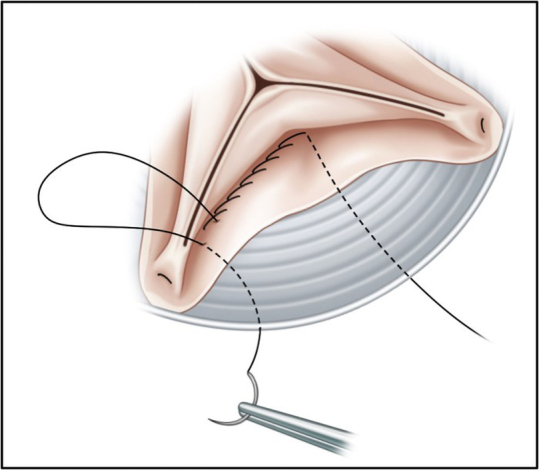
The half back stitch method was used to attach the native aortic valve inside the graft.

**Central Message**

The half back stitch is easy to adapt and offers efficient hemostatic lines during valve-sparing root reimplantation surgery.

Valve-sparing aortic root replacement (VSARR) offers many advantages over complete root replacement by avoiding the need for systemic anticoagulation and prosthetic-valve–related complications.^1^ Root reimplantation, the most widely accepted procedure in VSARR, may present some technical difficulties when the second-layer hemostatic stitches are made by conventional over-and-over continuous sutures within the woven-polyester graft—especially for beginning-level surgeons. Here we describe the half back stitch technique for the hemostatic suture line during the root reimplantation portion of VSARR (IRB #2024-0025; January 3, 2024, with a waiver of consent).

## Procedure

Cardiac arrest is induced with cardioplegia solution infusion after an aortic clamp placement. The aorta wall is resected leaving a margin of around 3 mm, coronary buttons are trimmed, and the root is completely mobilized. Acknowledging the argument over which is the best predictive model for choosing an adequate graft size during the root reimplantation, we follow our own simplified rule that the graft circumference should be shorter than the sum of the free-edge lengths of the cusps so that the leaflets can make the coaptation height. In most cases, graft selection was between 28 and 32 mm; body surface area was a minor consideration. Six nonpledgetted subannular stitches of 2–0 braided polyester are placed using horizontal mattress sutures that are then fixed at the bottom of the graft.

**Figure 1:**
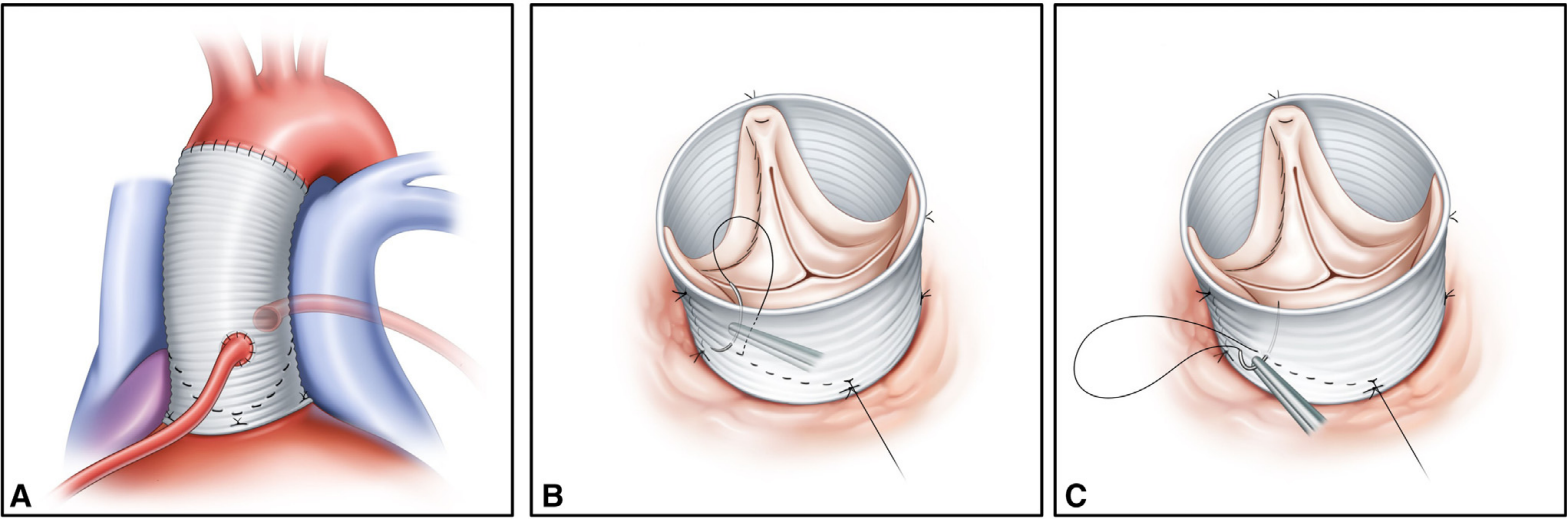
The half back stitch technique used in the modified version of valve-sparing aortic valve root reimplantation (*VSARR*). A, Final shape of the VSARR using the half back stitch technique B, Half back stitches were used to reattach the native aortic valve annulus inside the tubular graft. C, Hemostatic layer in the VSARR was made by half back stitches.

**Figure 2:**
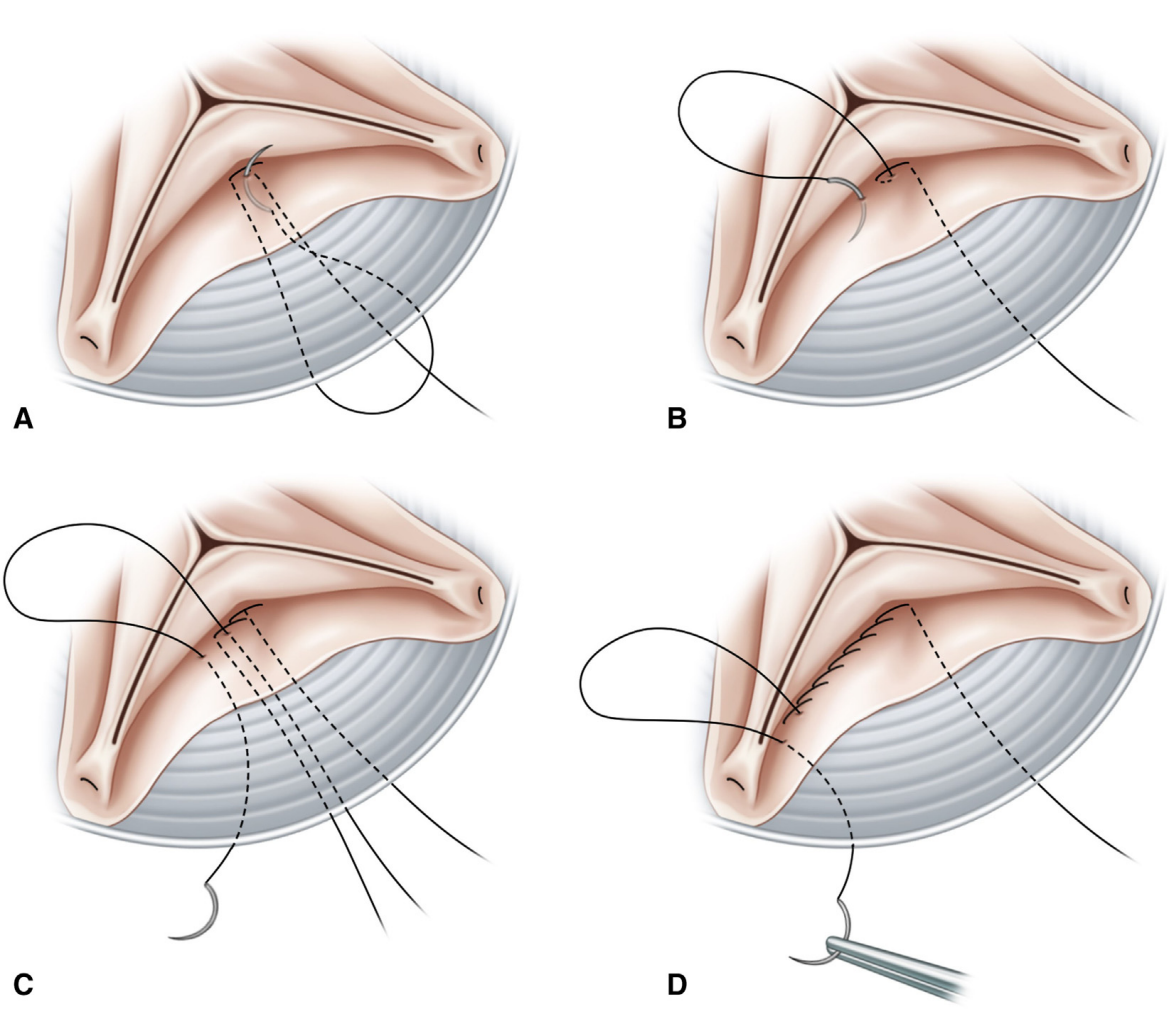
The procedural details of the half back stitch techniques in valve-sparing aortic valve root reimplantation (*VSARR*). A, Bilateral inside-out suture is placed at the bottom of the aortic valve annulus, and thereafter, the first half back stitch starts with the outside-in manner at the nadir. B, The next suture proceeds the inside-out way 10 mm distal to the first suture. C, The return suture goes via the outside-in manner 3 mm proximal to the prior suture. D, These returning sutures are repeated toward the commissure.

**Figure E1:**
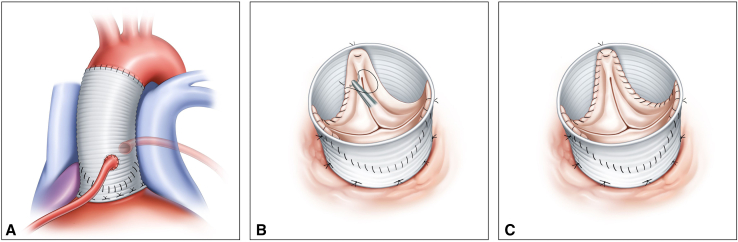
The whip stitch technique used in the standard manner of valve-sparing aortic valve root reimplantation (*VSARR*). A, Final shape of the standard VSARR. B, Continuous over-and-over stitches were used to reattach the native aortic valve annulus inside the tubular graft. C, Hemostatic layer in the standard VSARR was made by whip stitches.

The standard manner thereafter is to make hemostatic layer stitches to reattach the native aortic valve (AV) annulus inside the tubular graft using continuous over-and-over sutures referred to as the whip stitch technique in classic root reimplantation (Figure E1).^2^ We, however, have adopted a modified version of these attachment sutures; namely, the half back stitch (Figure 1). The aorta wall resection (remaining 3 mm) in the half back stitch technique is not different from standard root reimplantation using the whip stitch technique. First, the inside-out suture is performed at the nadir of AV annulus and it is tied down outside the graft (Figure 2, *A*). The next suture is started in an out-in manner. The following suture proceeds the inside-out way 10 mm distal to the first suture (Figure 2, *B*). Then, the return suture goes in the outside-in manner 3 mm proximal to the prior suture (Figure 2, *C*). After repeating this half back stitch, the thread is tied down outside of the graft at the commissure level (Figure 2, *D*). By repeating 3 sets of this continuous suture for each of the sinuses, the hemostatic layer is completed. When the leaflet prolapses, the central plication suture is added to elevate the coaptation of the corresponding leaflet. Thereafter, the coronary buttons are reattached to the graft. Finally, the distal part of the graft is anastomosed to the native aorta or another artificial graft depending on the extent of distal repair.

## Discussion

Implantation of the graft with AV preservation is an attractive option in aortic root surgery because it is believed to offer a lower risk of valve-related events compared with prosthetic valved graft conduit implantation.^1^ Society of Thoracic Surgeons data show that annual numbers of VSARR performance have been gradually escalating.^3^ During the past 2 decades, advancement in surgical techniques and reported excellent clinical outcomes by experienced hands support a merit of VSARR in aortic root replacement surgery.^4^

Nevertheless, beginning-level surgeons may feel barriers to performing the procedure because VSARR is time demanding and technically challenging.^5^ Among the several important technical steps in root reimplantation is constructing uniformly spaced hemostatic layers without wrinkling the graft within a safe timeline.

During the classic over-and-over continuous sutures (whip stitch) at the junction between the native aortic rim and the graft, a single motion of 180° vertical rotation of the needle along the suture lines that grasps both the native remnant aortic tissue and the graft is required. This is technically hard, but of paramount importance to avoid the architectural distortion that may result in serious bleeding and architectural distortion of the root geometry.^2^ On the contrary, the half back stitch involves straightforward penetration of each stitch because the suture line only exists in the overlapping area of the native aortic rim and the graft. In addition, the half back stitch may offer more stable hemostasis because it makes reinforced layers of suture lines. By the addition of a small backward stitch on each large forward stitch, it may also prevent purse-string shrinkage of the suture lines. We believe that these advantages may shorten the cardiac ischemic time and may help to construct effective hemostatic suture lines. In our institutional data, root implantation surgery using the half back stitch technique had significantly shorter procedure times (105 vs 155 minutes of cardiac ischemic time) compared with the whip stitch technique (Table 1). The result was consistent when the confounders (baseline profiles and combined procedures) were adjusted using propensity score matching. The half back stitch seems to shorten procedure times without significant deterioration in clinical outcomes.

**Table 1:** Clinical outcomes of the half back stitch technique compared with the classic root implantation technique

Variable	Overall cohort		
	Whip stitch (n = 105)	Half back stitch (n = 74)	*P* value
Characteristic			
Age (y)	46.8 ± 15.9	53.4 ± 14.7	.005
Male gender	78 (74.3)	61 (82.4)	.269
Body surface area	1.8 ± 0.2	1.9 ± 0.2	.054
Genetic aortopathy (%)	64 (61.0)	23 (31.1)	<.001
Bicuspid aortic valve	7 (6.7)	18 (24.3)	.002
Regurgitation grade			.31
None	27 (25.7)	11 (14.9)	
Mild or greater	78 (74.3)	63 (85.2)	
Severe	61 (58.1)	52 (70.3)	
Operations			
Combined valvuloplasty	69 (65.7)	45 (60.8)	.73
Maze	3 (2.9)	6 (8.1)	.22
Arch replacements	18 (17.1)	4 (5.4)	.03
Procedure time (min)			
Cardiopulmonary bypass	197.0 ± 65.4	130.2 ± 26.7	<.001
Cardiac ischemia	155.3 ± 49.8	105.1 ± 19.7	<.001
Postoperative aortic regurgitation			
Mild or greater	38 (36.3)	34 (46.0)	.30
Severe	0	2 (2.7)	.33
Early clinical outcomes			
Death	5 (4.8)	0	.15
Stroke	2 (1.9)	0	.64

Values are presented as n (%) or mean ± SD.

Some surgeons have described a running horizontal sewing technique for the hemostatic layer during root reimplantation. The horizontal running technique may be more difficult in hemostasis than in our technique because it is prone to shortening the suture, which results in a wrinkling of the sinus. We believe that the half back stitch technique prevents the purse-string effect because the 180° backward reinforcement suture may anchor the prior suture line.

In summary, the half back stitch technique is easy, timesaving, and hemostatic during VSARR (Video 1).

## Conflict of Interest Statement

The authors reported no conflicts of interest.

The *Journal* policy requires editors and reviewers to disclose conflicts of interest and to decline handling manuscripts for which they may have a conflict of interest. The editors and reviewers of this article have no conflicts of interest.

## Supplementary Data

Video 1 Valve-sparing aortic valve root reimplantation was performed using the half back stitch technique to attach the native aortic valve in a tubular graft. Video available at: https://www.jtcvs.org/article/S2666-2507(24)00074-9/fulltext.
