# Supplementary figures and images for: Half back stitch for root reimplantation
Source: JTCVS Tech. 2024 Apr 25;25:19–23. doi: 10.1016/j.xjtc.2024.02.017 (PMC11184480; doi:10.1016/j.xjtc.2024.02.017)

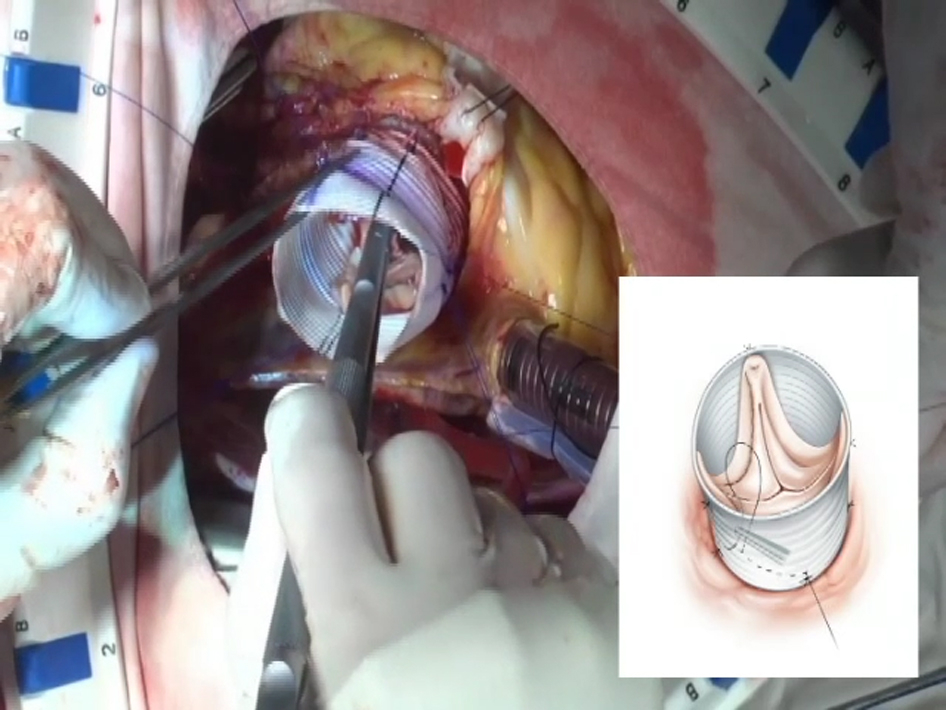

Supplement: Video 1 — Valve-sparing aortic valve root reimplantation was performed using the half back stitch technique to attach the native aortic valve in a tubular graft. Video available at: https://www.jtcvs.org/article/S2666-2507(24)00074-9/fulltext. [file fx2.jpg]
